# Cytokines and Depressive Symptoms Among Adolescents

**DOI:** 10.1177/10998004251318385

**Published:** 2025-02-04

**Authors:** Cherry Y. Leung, Sandra J. Weiss

**Affiliations:** 1 Department of Community Health Systems, School of Nursing, 8785 University of California San Francisco, San Francisco, CA, USA

**Keywords:** cytokines, adolescence, inflammatory biomarkers, depression, depressive symptoms

## Abstract

**Background:** Inflammation has been linked to an increased risk of depression, but there is limited and conflicting research on the role of inflammatory markers in adolescent depression. The purpose of this study was to examine associations between cytokines TNF-α, IL-1β, IL-6, and IL-8 and depression among a community-based sample of adolescents (13–19 years of age). **Methods:** Salivary samples were self-collected by adolescents for assay of cytokines. The Patient Health Questionnaire-9 (PHQ-9) was used to measure depressive symptoms and clinical depression, where a score ≥11 indicated the threshold for experiencing clinical depression. Multiple linear and logistic regression models were used to examine the relationships between cytokines and depression, adjusting for age, sex, ethnicity, income, and body mass index. **Results:** The mean age of the 83 participants was 15.86 years. Eight participants screened positive for depression; the mean depressive symptom score was 5.11. Higher levels of IL-6 (Coef = 1.33, *p* < .001) and IL-8 (Coef = 0.69, *p =* .025) were associated with more frequent depressive symptoms while higher levels of TNF-α (OR = 2.50, *p =* .002), IL-1β (OR = 1.98, *p* = .001), and IL-8 (OR = 2.44, *p* = .008) were associated with greater odds of meeting criteria for clinical depression. **Conclusions:** Future research should focus on factors that induce higher cytokine levels and the mechanisms underlying their effects on depression. Cytokines assessed in this study may ultimately have implications as methods for depression screening or targets for biologic interventions to prevent and treat adolescent depression.

## Introduction

Adolescence is a critical period of development marked by social and emotional changes that heighten vulnerability to depression. Adolescent depression is a major public health concern as it is a leading cause of illness and disability worldwide (Petito et al., 2020; World Health Organization, 2024). Studies estimate that around 5% of young adolescents experience diagnosed depression, with that number rising to 20% in late adolescence (Petito et al., 2020). However, since many depressed teens are never identified by their clinician as having depression, these figures likely underestimate the true scope of the problem (Zuckerbrot et al., 2018). Many adolescents grapple with depressive symptoms that fall below the diagnostic threshold for depression, and a significant portion remains undiagnosed or inadequately treated (Cuijpers et al., 2021; Noyes et al., 2022). The consequences of depression and depressive symptoms can be severe, affecting academic achievement (Verboom et al., 2014), social interactions, and increasing the risk of substance use (Boers et al., 2019; Twenge & Campbell, 2018) and suicide (Grossberg & Rice, 2023; U. S. Preventive Services Task Force et al., 2022). Furthermore, untreated depressive symptoms in adolescence can significantly increase the risk of developing chronic depression in adulthood (Johnson et al., 2018). While numerous risk factors contribute to adolescent depression, the potential role of inflammation remains an understudied area.

There is growing evidence that immune dysregulation and associated inflammatory mechanisms may play a role in the pathogenesis of depressive symptomology (D’Acunto et al., 2019). For example, Kappelmann et al. (2018) reported that individuals with depression often have elevated inflammatory cytokines, that patients with inflammatory conditions are at increased risk for depression, and that cytokine-based treatments—therapies designed to modulate the immune response using cytokines—may lead to depression. Other studies have shown that increased inflammation decreases anti-depressant medication response (D’Acunto et al., 2019; Leighton et al., 2018) and early-life infection and autoimmune diseases are associated with higher depression risk (Benros et al., 2013; Euesden et al., 2017; Leone et al., 2022). A meta-analysis of 107 studies in the general adult population found that individuals with depression had elevated C-reactive protein (CRP), interleukin (IL)-3, IL-6, IL-12, IL-18, soluble interleukin 2 receptor (sIL-2R), and tumor necrosis factor alpha (TNF-α) levels with medium-effect sizes (Osimo et al., 2020). On the other hand, Haapakoski et al. (2015) in their meta-analysis reported that while higher IL-6 levels were associated with depression, there were no consistent associations between IL-1β and TNF-α with depression. Other studies have shown that alterations in cytokine levels are associated with anti-depressant treatments. For example, adult patients with Major Depressive Disorder (MDD) who responded to anti-depressants had lower baseline IL-8 levels and decreased TNF-α levels compared to non-responders (Liu et al., 2020).

There are several systematic reviews (Mills et al., 2013; Schumacher et al., 2024) and meta-analyses (Colasanto et al., 2020; D’Acunto et al., 2019) focused on inflammation and depression in the adolescent population, but few studies met the criteria for the reviews and these studies were either cross-sectional, had small sample sizes, or did not cover a range of inflammatory biomarkers. Moreover, while there is evidence that inflammation is associated with adolescent depression, results are mixed. In a recently published meta-analysis on case-control studies of inflammatory cytokines and depression, participants with depressive disorders had a trend toward significantly higher TNF-α (D’Acunto et al., 2019). Another review examining the role of cytokines in adolescence found that IL-1β and TNF-α were associated with an increased risk of depression, and this relationship was influenced by neurodevelopment, hormonal changes, stress, and trauma (Mills et al., 2013). A more recent meta-analysis by Colasanto et al. (2020) consisted of 22 studies conducted on children and adolescents and found bi-directional influences where depression was a predictor of IL-6 while both IL-6 and CRP predicted future depression. Lastly, the levels of a lesser-studied pro-inflammatory cytokine, IL-8, were reported to be lower in the MDD group versus healthy control (HC) group at baseline in a group of adolescents and young adults (Skibinska et al., 2022).

Additionally, the relationship between inflammation and severity of symptoms is understudied, because most research has examined how inflammation relates to an actual diagnosis of depression or to a clinical cutoff on a screening tool that suggests the need for a diagnostic work-up. Thus, there is the need to not only study how inflammatory markers may characterize adolescents who meet the criteria for depression but also the relationship of these markers to degrees of depression severity on a symptom scale. Our study aimed to 1) describe the prevalence of depression among a community sample of adolescents and the severity of their depressive symptoms, and 2) examine the associations of pro-inflammatory biomarkers TNF-α, IL-1β, IL-6, and IL-8 with both severity of depressive symptoms and an established clinical cutoff for depression that suggests a need for diagnostic referral.

## Methods

### Sample and Procedures

Adolescents 13–19 years of age were recruited from an adolescent primary care clinic affiliated with the UCSF Medical Center and through advertisements posted in public places (e.g., libraries, coffee shops, and on Facebook groups) on the Bay Area, United States (U.S.). Adolescents interested in the study were introduced to our study in person, via email or by phone. A research assistant (RA) provided detailed information about the study and obtained informed assent from the adolescent and consent from the parent if the adolescent was under 18 years of age. Informed consent was obtained for the adolescent 18–19 years of age. Adolescents were excluded from participation if they had been on medications for depression within the previous six months, had co-morbid mental health conditions (with the exception of anxiety), were on oral steroids, or had a prior diagnosis of an inflammatory condition. The study was approved by the Institutional Review Board of the University. Following assent and consent, participants completed depressive symptom and sociodemographic questionnaires and provided salivary samples either in the clinic or at their homes. Recruitment occurred between February 2018 to June 2020. Details of data collection have been reported elsewhere (Leung et al., 2024).

### Salivary Cytokine Samples

Participants received saliva collection kits at recruitment or through the mail, along with verbal and written instructions about collection and storage. They were asked not to consume food or exercise for 1 hour before providing their saliva sample and to rinse their mouths with water 10 minutes before providing the sample. The adolescents then provided 1 mL of saliva through the passive drool method using SalivaBio’s 2 mL cryovials and collection aids (Salimetrics, State College, PA, https://www.salimetrics.com). Participants were instructed to open the collection vial, insert the ribbed end of the Saliva Collection Aid into the vial, allow saliva to naturally pool in their mouth, then gently guide the saliva into the vial by tilting their head forward and letting it passively drool through the collection aid. When the indicator on the vial showed 1 mL of saliva, they stopped sample provision and capped the vial.

Following collection of each sample, the participants were instructed to place the sample in the freezer and contact our research team for pick-up. Samples were frozen at −80°C and stored in a Clinical Research Services laboratory until they were ready for analysis. Samples were assayed at Salimetrics Bioscience Clinical Laboratory using a sandwiched enzyme immunoassay (ELISA) following the Salimetrics protocol (Salimetrics, n.d). Samples were assayed for the Salimetrics Cytokine Panel (TNF-α, IL-1β, IL-6, and IL-8) and analyzed in duplicate at the Salimetrics SalivaLab (Carlsbad, CA) using a proprietary electrochemiluminesence method developed and validated for saliva by Salimetrics. The average coefficient of variation for all samples tested was <15%, which meets the SalivaLab’s criteria for accuracy and repeatability in Salivary Bioscience, and exceeds the applicable National Institute of Health (NIH) guidelines for Enhancing Reproducibility through Rigor and Transparency (NIH, 2024). Sample test volume was 25 μL of saliva per determination. The assay has a lower limit of sensitivity of 0.0314 pg/mL, (TNF-α), 0.0195 pg/mL (IL-1β), 0.0491 pg/mL (IL-6), and 0.0201 pg/mL (IL-8), with a dynamic range from 0.0314–380 pg/mL (TNF-α), 0.0195–589 pg/mL (IL-1β), 0.0491–736 pg/mL (IL-6), and 0.0201–574 pg/mL (IL-8).

### Self-Reported Depression Scores

The Patient Health Questionnaire-9 (PHQ-9) was used to assess the severity of depressive symptoms and determine whether the adolescents met the clinical cutoff for depression. Higher scores were associated with worsening functional status and increased disability days, and scores ≥10 had a sensitivity of 83% and a specificity of 89% for clinical cut-off of major depression for adults >18 years of age. The PHQ-9 has also shown good specificity (89.5%) and sensitivity (77.5%) among U.S. adolescents (13–17 years of age) (Richardson et al., 2010). Scores ≥11 had a sensitivity of 89.95% and specificity of 77.5% for clinical cut-off of major depression for adolescents (Richardson et al., 2010), showing promising results that the PHQ-9 can accurately measure depression symptom severity in adolescents with depression (Croarkin et al., 2021). Among adolescents, the PHQ-9 showed excellent internal consistency (Cronbach’s α = 0.92) (Monga et al., 2024) and good convergent validity with the Beck’s Depression Inventory-II (correlation = 0.88) (Bentley et al., 2021).

Participants rated how frequently they had experienced depressive symptoms over the past two weeks on a scale ranging from 0 to 3 (0 for “not at all” and 3 for “nearly every day”). Total scores range from 0 to 27 points, with higher scores indicating more depressive symptoms (Kroenke et al., 2001). Scores are also used to classify individuals as having depression that is minimal (<5), mild (5–9), moderate (10–14), moderately severe (15–19), or severe (20–27). A *cut-*off score of ≥11 was used to designate clinical depression in our study.

The Principal Investigator of the study, a Board Certified Psychiatric Mental Health Nurse Practitioner, with expertise in child and adolescent mental and behavioral health, was notified if participants scored ≥11 on the PHQ-9 or displayed any concerns of harm to self or others. Based on this appointment, a needs assessment was made. All these participants were provided with mental health resources specific to their needs and referred to their primary care clinician and/or a mental health care provider for further assessment. Parents were notified if participants were <18 years of age.

### Confounders

Potential confounding factors consistently shown to influence the relationship between inflammation and depression, as identified by the literature (Colasanto et al., 2020), were included in our models. Confounders we examined included participant age (13–19 years), sex (self-reported male or female), ethnicity (Hispanic or Not), and income (<75,000, $75,000–$100,000, $101,000–$150,000, and > $150,000). Additionally, body mass index (BMI, kg/m^2^) was considered a potential confounder, having been reported to be associated with both inflammation and depression (Palmos et al., 2023). Effects of race were not considered in our analysis due to the small number of participants in different racial groups.

### Sample Size

Mplus (v7.4) was employed to determine sample size, using 10,000 Monte Carlo simulations (Muthén & Muthén, 1988-2017, 2002). We estimated the ability to detect a medium effect size based on results of the most comprehensive meta-analysis to date that has looked at relationships between depression and cytokines (Osimo et al., 2020). We found that for a two-sided test with alpha = 0.05, we could detect a standardized effect of 0.504 for our continuous independent variables (cytokines) and our continuous dependent variable (depressive symptoms) with a sample size of 90.

### Data Analysis

Sample characteristics were examined with descriptive statistics. Multiple imputation was used to handle missing data (ethnicity 3.8%, race 2.4%, and household income 3.8%), with 10 imputed datasets created. Due to the right skew of our pro-inflammatory cytokine levels, TNF-α, IL-1β, IL-6, and IL-8 were log transformed. We used multiple linear regression models to examine the associations between the four pro-inflammatory cytokines (TNF-α, IL-1β, IL-6, and IL-8) and depressive symptoms. Unstandardized beta coefficients (Coef), standard errors (SE), *p*-values (*p*), 95% confidence intervals (CIs), and standardized betas (β) are reported. These models adjusted for age, sex, ethnicity, household income, and BMI. We used multiple logistic regression models to examine the associations between pro-inflammatory cytokines (TNF-α, IL-1β, IL-6, and IL-8) and the cutoff for clinical depression. Odds ratios (ORs), SE, *p*-values, and 95% CI are reported. These models adjusted for age, ethnicity, household income, and BMI. Sex was considered in the logistic regression models, but was ultimately excluded due to wide 95% CIs, indicating an imprecise estimation of its effect on the outcome. We analyzed the data using STATA (Version 18). We evaluated all tests of significance with a two-sided α of 0.05.

## Results

Eighty-three participants were included in this study as they had complete data for both the exposure and outcome variables (Table 1). The mean age for the sample was 15.86 years (standard deviation [SD] = 1.77). This overall sample was racially and ethnically diverse, including 47% non-white teens and approximately 32% Hispanic teens; however, as previously described, race was not included in our final models because the specific number of participants within each racial category was insufficient for robust statistical analysis. The largest percent of teens were in an income range between $101,000–$150,000 (42.0%), which would be considered middle income ($122,000) in the region where the study occurred as compared to the national middle income of $76,330 (U.S. Census Bureau, n.d). Mean pg/dl levels for the 4 pro-inflammatory cytokines were 338.13 (IL-1β), 9.52 (IL-6), 1188.47 (IL-8), and 3.86 (TNF- α). The mean PHQ-9 score for this sample was 5.11. Eight participants had a PHQ-9 score ≥11 (cutoff for clinical depression) while 75 participants had a score <11. Of those who had PHQ-9 scores that met the cut-off for clinical depression, six were ≥16 years of age, five were female, and three were overweight based on their BMI scores.

**Table 1.** Demographic and Clinical Data for Participants in the Study (*n* = 83)^a^.

Variables	Mean (SD, Range) or *n* (%)
Age (years)	15.86 (1.77, 13–19)
Sex	
Male	42 (50.6)
Female	41 (49.4)
Race^b^	
White	43 (53.1)
Black	5 (6.2)
Asian	17 (21.0)
Other	3 (3.7)
Multi-racial	13 (16.1)
Ethnicity^c^	
Not Hispanic	54 (67.5)
Hispanic	26 (32.5)
Household income^b,c^	
<$75,000	20 (24.7)
$75,000–$100,000	12 (14.8)
$101,000–$150,000	34 (42.0)
>$150,000	15 (18.5)
BMI	23.11 (4.29, 14.40–33.30)
Pro-inflammatory cytokines^d^	
IL-1β	340.66 (708.25, 2.65–3436.01)
IL-6	9.52 (28.95, 0.69–257.28)
IL-8	1188.47 (1182.64, 43.73–4435.60)
TNF-α	3.86 (4.94, 0.16–32.46)
PHQ-9	
Depressive symptoms	5.11 (4.24, 0–23)
Depression	
No	75 (90.4%)
Yes	8 (9.6%)

*Note.* SD, standard deviation; number; %, percentage; BMI, body mass index (lbs/inches^2^); PHQ-9, Patient Health Questionnaire-9.

^a^Total sample size of study with exposure and outcome variables.

^b^Missing data: Race (2.4%), ethnicity (3.8%), income (3.8%).

^c^USD.

^d^SD out of range due to the right skew of the cytokine data.

Tables 2 and 3 show our results for the associations between pro-inflammatory cytokines IL-6 and IL-8 and depressive symptoms. IL-6 was associated with more depressive symptoms (Coef = 1.33, *p* < .001) after adjusting for age, sex, ethnicity, income, and BMI. Similarly, the adjusted model indicated that IL-8 was associated with more depressive symptoms (Coef = 0.69, *p =* .025). There were no statistically significant associations between either TNF-α or IL-1β and depressive symptoms (see Supplemental Tables 1 and 2).

**Table 2.** Linear Regression^a^ Model Examining the Associations Between Pro-Inflammatory Cytokine IL-6 and Depressive Symptoms.

Exposure	Outcome				
	Depressive Symptoms				
	Coef	SE	*p*-Value	95% CI	β
IL-6	1.33	0.32	<.001	0.69 to 1.97	0.33
Sex					
Male	Ref				
Female	−1.61	0.62	.010	−2.84 to −0.38	−0.22
Age	0.12	0.17	.923	−0.32 to 0.35	0.01
Ethnicity					
Not Hispanic	Ref				
Hispanic	−0.71	0.72	.324	−2.12 to 0.71	−0.09
Household income					
<$75,000	Ref				
$75,000–$100,000	−0.13	0.99	.897	2.08 to 1.83	−0.01
$101,000–$150,000	1.60	0.73	.031	0.15 to 3.05	0.22
>$150,000	1.51	1.03	.144	−0.52 to 3.55	0.12
BMI	0.06	0.08	.426	−0.09 to 0.21	0.07

*Note.* Coef, unstandardized beta coefficient; SE, standard error; CI, confidence interval; β, standardized beta coefficient.

^a^Adjusted for sex, age, ethnicity, household income, and BMI.

**Table 3.** Linear Regression^a^ Model Examining the Associations Between Pro-Inflammatory Cytokine IL-8 and Depressive Symptoms.

Exposure	Outcome				
	Depressive Symptoms				
	Coef	SE	*p*-Value	95% CI	β
IL-8	0.69	0.30	.025	0.09 to 1.29	0.17
Sex					
Male	Ref				
Female	−1.55	0.64	.017	−2.82 to −0.28	−0.21
Age	0.01	0.18	.965	−0.34 to 0.35	0.004
Ethnicity					
Not Hispanic	Ref				
Hispanic	−0.33	0.73	.653	−1.78 to 0.71	0.04
Household income					
<$75,000	Ref				
$75,000–$100,000	0.39	1.04	.711	−1.67 to 2.45	0.03
$101,000–$150,000	1.95	0.76	.053	−0.19 to 2.98	0.20
>$150,000	1.75	1.08	.108	−0.39 to 3.88	0.14
BMI	−0.34	0.07	.645	−0.18 to 0.11	−0.04

*Note.* Coef, unstandardized beta coefficient; SE, standard error; CI, confidence interval; β, standardized beta coefficient.

^a^Adjusted for sex, age, ethnicity, household income, and BMI.

Tables 4–6 show the results for the 3 significant associations between cytokines and the cutoff for clinical depression. While there were no statistically significant associations between IL-6 and depression (see Supplemental Table 3), IL-1β (OR = 1.98, *p =* .001), IL-8 (OR = 2.44, *p =* .008), and TNF-α (OR = 2.46, *p =* .002) were associated with a greater odds of having depression, adjusting for age, ethnicity, income, and BMI.

**Table 4.** Logistic Regression^a^ Model Examining the Associations Between Pro-Inflammatory Cytokine IL-1β and PHQ-9 Depression.

Exposure	Outcome			
	Depression			
	OR	SE	*p*-Value	95% CI
IL-1β	1.98	0.41	.001	1.32 to 2.97
Age	1.72	0.29	.001	1.24 to 2.40
Ethnicity				
Not Hispanic	1			
Hispanic	4.63	2.61	.007	1.53 to 13.97
Household income				
<$75,000	1			
$75,000–$100,000	1.74	1.63	.554	0.28 to 10.94
$101,000–$150,000	1.37	0.92	.640	0.37 to 5.13
>$150,000	1.38	1.43	.757	0.18 to 10.60
BMI	1.02	0.07	.763	0.90 to 1.16

*Note.* PHQ-9, Patient Health Questionnaire-9; OR, odds ratio; CI, confidence interval.

^a^Adjusted for age, ethnicity, household income, and BMI.

**Table 5.** Logistic Regression^a^ Model Examining the Associations Between Pro-Inflammatory Cytokine IL-8 and PHQ-9 Depression.

Exposure	Outcome			
	Depression			
	OR	SE	*p*-Value	95% CI
IL-8	2.44	0.83	.008	1.26 to 4.74
Age	1.74	0.28	.001	1.27 to 2.39
Ethnicity				
Not Hispanic	1			
Hispanic	5.90	3.31	.002	1.97 to 17.69
Household income				
<$75,000	1			
$75,000–$100,000	1.67	1.54	.577	0.28 to 10.13
$101,000–$150,000	0.98	0.62	.971	0.28 to 3.40
>$150,000	1.11	1.21	.926	0.13 to 9.37
BMI	1.02	0.07	.763	0.90 to 1.15

*Note.* PHQ-9, Patient Health Questionnaire-9; OR, odds ratio; CI, confidence interval.

^a^Adjusted for age, ethnicity, household income, and BMI.

**Table 6.** Logistic Regression^a^ Model Examining the Associations Between Pro-Inflammatory Cytokine TNF-α and PHQ-9 Depression.

Exposure	Outcome			
	Depression			
	OR	SE	*p*-Value	95% CI
TNF-α	2.50	0.75	.002	1.39 to 4.51
Age	1.68	0.28	.002	1.22 to 2.33
Ethnicity				
Not Hispanic	1			
Hispanic	4.93	2.76	.004	1.64 to 14.8
Household income				
<$75,000	1			
$75,000–$100,000	2.06	1.83	.417	0.36 to 11.73
$101,000–$150,000	1.15	0.75	.826	0.32 to 4.14
>$150,000	1.24	1.27	.832	0.17 to 9.24
BMI	1.01	0.06	.891	0.89 to 1.14

*Note.* PHQ-9, Patient Health Questionnaire-9; OR, odds ratio; CI, confidence interval.

^a^Adjusted for age, ethnicity, household income, and BMI.

Results show significant effects for some of the covariates as well. In both adjusted linear regression models (Tables 2 and 3), females, compared to males, were at lower risk for having depressive symptoms (IL-6 model: female Coef = −01.61, *p =* .010; IL-8 model: female Coef = −1.55; *p =* .017). Household income $100,000–150,000 compared to < $75,000 had higher risk for depressive symptoms in the IL-6 model ($101,000–$150,000 Coef = 1.60, *p =* .031) while there was a trend towards significance in the IL-8 model ($101,000–$150,000 Coef = 1.95, *p =* .053). For all three adjusted logistic regression models, increasing age and Hispanic, compared to Not Hispanic, teens were at higher risk for PHQ-9 depression (see Tables 4–6).

## Discussion

Most adolescents in the sample scored in the range of mild depressive symptoms (5–9) (Kroenke et al., 2001). Six of the eight adolescents who screened positive for clinical depression on the PHQ-9 were over 16 years of age, suggesting that the prevalence of depression increases among older adolescents (14%, 16–19 years) compared to younger adolescents (5.9%, 13–15 years). These findings align with previous reports on prevalence of depression among adolescents (Merikangas et al., 2010; World Health Organization, 2024). Additionally, our study found that more females (*n* = 5) than males (*n* = 3) met the cut-off for PHQ-9 clinical depression, findings which are consistent across many studies where depression is reported to be more prevalent among females (Albert, 2015). At the same time, the 2021 National Survey on Drug Use reported that 29.2% of female adolescents had a major depressive episode compared to 11.5% of male adolescents (U.S. Department of Health and Human Services et al., 2022). Our results, in concert with these other findings, indicate the need for more research on causative factors underlying gender differences in teen depression.

Across all the inflammatory markers we examined, higher levels of cytokines were associated with greater depression. However, some cytokines were linked to increasing symptom severity while others appeared to predict the adolescents’ odds of meeting criteria for potential clinical depression. IL-8 had the most consistent association with depression, predicting both increases in symptom severity and greater odds of meeting criteria for clinical depression. In separate linear regression models that adjusted for multiple covariates, higher levels of IL-6 and IL-8 were associated with increasing levels of depressive symptoms, with the odds of meeting criteria for clinical depression being nearly 2.5 times greater with every unit (pg/dL) increase in IL-8. For every unit (pg/dL) increase in IL-1β, the odds of clinical depression rose by 98%. Lastly, for every unit increase (pg/dL) in TNF- α, the odds of clinical depression rose approximately 2.5 times or by 150%.

### IL-6 and Depressive Symptoms

Our finding that increasing IL-6 levels were associated with increasing severity of depressive symptoms may be explained by the effect of IL-6 on biological systems previously linked to depression. Research suggests that increased IL-6 contributes to the disruption of neurotransmitter metabolism (e.g., decreased serotonin) which can alter synaptic neurotransmission, dysregulation of the hypothalamic-pituitary-adrenal (HPA) axis, and reduction of neurotrophic factors such as brain-derived neurotrophic factor (BDNF), all of which can lead to depressive symptoms (Mikulska et al., 2021; Ting et al., 2020). IL-6 exerts its effects by activating immune cells, which can disrupt the blood-brain barrier, and negatively impact neuronal function and mood regulation (Ting et al., 2020). However, we did not observe an association between IL-6 and the likelihood of developing clinical depression. This suggests that while IL-6 may be involved in the frequency of experienced symptoms, it may not be a marker of vulnerability to full-blown clinical depression. It is possible that short-term fluctuations in IL-6 are related to episodic symptoms of depression but that they do not persist and foster chronic inflammation. Less chronic inflammation would likely contribute to short-lived alterations in neurotransmitters that could influence depression (Kummer et al., 2021; Tanaka et al., 2014), resulting in effects not persistent or severe enough to meet the criteria for a clinical depression diagnosis.

Significant knowledge gaps remain regarding the role which IL-6 plays in adolescent depression. Further research is needed to explore the specific mechanisms by which IL-6 modulates depressive symptom severity. Since our assessment of IL-6 was a one-time measure, it will also be particularly important to examine how changes in IL-6 throughout the day and longitudinally, parallel concurrent changes in the adolescents’ depressive symptoms. Assessing the interplay between IL-6 levels and treatment response could offer valuable insights as well into its potential and its limitations as a therapeutic target for depression.

### TNF-α and IL-1β as Potential Markers of Clinical Depression

In contrast to IL-6, TNF-α and IL-1β levels were both associated with an increased odds of actually meeting criteria for clinical depression. These findings align with existing research highlighting the role of these cytokines in stress response dysregulation and altered neuroplasticity, both of which are implicated in depression (Hassamal, 2023). TNF-α and IL-1β share some functional similarities, potentially acting through overlapping pathways (Alvarez et al., 2020; Bonaventura et al., 2018). Previous research has shown also that TNF-α and IL-1β disruption affects depression by hindering serotonin production and signaling, a crucial neurotransmitter for mood regulation (Das et al., 2021; Han & Yu, 2014; Ng et al., 2018). These two cytokines can also activate an enzyme indoleamine 2,3-dioxygenase (IDO) that further depletes serotonin by creating the byproduct kynurenine, resulting in the development of depressive symptoms (Haq et al., 2021; Liu et al., 2015). As proteins that regulate activity of the immune system, higher levels of TNF-α and IL-1β contribute to neuroinflammation, typically a low-grade inflammatory state that damages neurons and disrupts brain circuits involved in mood (Hassamal, 2023).

Furthermore, research has shown that the negative impact of IL-1β extends to synaptic plasticity (Hoshino et al., 2017), the brain’s ability to adapt and strengthen new connections between neurons. This impaired neuroplasticity may be particularly important for the adolescents’ developing brain. It could hinder the teen’s ability to cope with stress and negative emotions, potentially worsening symptoms of depression. However, further investigation is needed to elucidate their distinct contributions in clinical depression. Exploring their potential interactions or how they differ from IL-6 in modulating a more severe state of clinical depression rather than only the frequency of depressive symptoms could provide valuable insights for future research and clinical practice.

### IL-8 as a Distinctive Biomarker of Depression

Our study yielded a unique finding regarding IL-8. Unlike the other cytokines, higher IL-8 levels were associated with both the severity of depressive symptoms and twice the odds of developing actual clinical depression. These findings suggest a potentially significant role for IL-8 in development of adolescent depression. A more substantial role for IL-8 in depression could be due to a variety of factors. First, IL-8 is synthesized and released early during the inflammatory response phase and can persist for days or weeks, rather than the transient effects of other cytokines, where they are cleared within hours (Remick, 2005). This early and sustained response could result in more potent and longer-term effects of IL-8 on depression. Second, IL-8 may mediate the pathway between pervasive factors such as life stress and depression (Mills et al., 2013; Szelenyi & Vizi, 2007), increasing ongoing risk for development of depressive symptoms. These symptoms, in turn, could further exacerbate the inflammatory response and elevate inflammatory biomarker levels, creating a cycle that increases the risk of depression (Kiecolt-Glaser et al., 2015). Third, research indicates that IL-8 may directly influence brain regions and neurotransmitter systems related to mood regulation, contributing to depressive symptoms through multiple mechanisms (Tsai, 2021). Lastly, IL-8 might also interact with other inflammatory markers (Bester & Pretorius, 2016) or cellular processes (Bernhard et al., 2021) that ultimately lead to depression onset.

Findings for the role of IL-8 in adult depression are conflicting, with some research indicating increased IL-8 levels among depressed females but not depressed males (Birur et al., 2017) and lower IL-8 levels among medication naïve patients with MDD compared to controls (Cakici et al., 2020). The one study that examined IL-8 in adolescent depression reported opposite findings to ours, in that MDD patients had lower IL-8 levels at baseline compared to healthy controls (Skibinska et al., 2022). In another study involving a community sample of adolescents, lower IL-8 levels also predicted more depressive symptoms in males, but not females (Moriarity et al., 2019). Differences between those studies and ours may be due to study design and methodology. Our analysis incorporated additional and/or different confounding variables than Skibinska et al. (2002) and Moriarity et al. (2019). Skibinska et al. (2022) adjusted for age and sex while Moriarity et al. (2019) adjusted for sex, age, BMI, and family income and focused on a specific population (Caucasians, African Americans, and Biracial teens). Additionally, differences in the heterogeneity of sample characteristics could contribute to these observed variations. Skibinska et al.’s (2022) study involved older Polish adolescents (mean age = 18.49 years, SD = 3.2), with clinically diagnosed MDD and bipolar disorder compared to healthy controls. Moriarity et al.’s (2019) study consisted of a community sample of youth 12–13 years of age specifically examining differences among specific racial groups. Pubertal changes in sex hormones may also play a role in the pathogenesis of adolescent depression, as indicated by the sex-based differences in Moriarity et al.’s (2019) results. More research is needed to fully elucidate the specific mechanisms by which IL-8 exerts its influence on both depressive symptoms and the development of clinical depression among adolescents. This should include evaluation of biological, social and psychological mechanisms. Investigating how IL-8 levels change over time in the presence or absence of depression treatment could also provide valuable information regarding this biomarker as a potential therapeutic target for intervention.

### Strengths and Limitations

This study has many strengths, including the examination of four different pro-inflammatory cytokines, including the understudied IL-8, and their relationships with both the established cutoff for clinical depression and the increasing severity of depressive symptoms among a group of adolescents in a community setting. We controlled for the key demographic variables of age, sex (linear regression models), ethnicity, and income. Lastly, our study utilized non-invasive, salivary biospecimens, which are both correlated with serum blood levels (Byrne et al., 2013; La Fratta et al., 2018) and found by some research to be a more optimal peripheral fluid to assess inflammatory processes (Parkin et al., 2023).

On the other hand, the study had several limitations. Due to the cross-sectional design, we cannot establish causality and examine the long-term effects of cytokines and depression. For instance, unlike acute inflammation associated with injuries, a constant and chronic, low-grade inflammatory state disrupts various bodily processes. Pro-inflammatory cytokines released during chronic inflammation (Koelman et al., 2019) can disrupt the production of neurotransmitters like serotonin and brain-derived neurotrophic factor (BDNF), both of which are essential for mood regulation and brain plasticity (Porter & O’Connor, 2022; Rhie et al., 2020). We were not able to examine the trajectory of these developmental processes. Still, understanding the potential role of inflammation in adolescent depression opens doors for future research and more targeted treatment approaches. Next, as previously described, we considered potential confounding factors in our models, but the list is not exhaustive as overfitting the models can lead to inaccurate estimates. For example, our study neither controlled for sex hormones nor pubertal status, a proxy measurement for adolescent development, which can affect the immune system and inflammation (Ucciferri & Dunn, 2022). We also did not control for physical activity, a lifestyle factor that may both decrease inflammation and depressive symptoms (Kandola et al., 2019). At the same time, utilizing a sample of adolescents with several exclusion criteria has its strengths as fewer potential confounds (e.g., medications, other mental health conditions) affect the inflammatory measures (Moriarity et al., 2019). We measured depression using self-reported surveys rather than clinician diagnosis, the gold standard. Therefore, it is not known whether the relationships we found between cytokines and self-reported depression would be replicated with a clinical diagnosis of depression. Due to the small number of adolescents screening positive for clinical depression in our study, we had decreased power to detect significant effects. Future studies should consider oversampling these individuals. A limitation of this approach, however, is that adolescents having been diagnosed with depression are likely to be on anti-depressant medications, which may confound results. Lastly, our small sample size overall increases the chance of inflated estimates and wider confidence intervals with less precise estimates. Nonetheless, this study provides new knowledge to inform future research.

## Conclusions

Our findings suggest that various inflammatory biomarkers are associated with adolescent depression and its symptoms. Although effect sizes were small, higher levels of TNF-α, IL-1β, IL-6, and IL-8 were all linked to either more depressive symptoms and/or greater odds of meeting criteria for clinical depression. IL-8 appeared to play a role in both severity of depressive symptoms and a higher risk for full-blown clinical depression, indicating a particular need for further research on factors that induce higher IL-8 levels and the mechanisms underlying their effects. IL-8 and the other cytokines we studied may ultimately have implications as methods in screening for depression or targets for biologic interventions to prevent and treat adolescent depression. The inclusion of inflammatory biomarker testing in diagnostic assessments could offer valuable insights, potentially facilitating earlier diagnosis and treatment initiation. Additionally, future studies exploring chronic inflammation are essential to better understand its role in the development and progression of depression.

## Supplemental Material

Supplemental Material - Cytokines and Depressive Symptoms Among Adolescents Supplemental Material for Cytokines and Depressive Symptoms Among Adolescents by Cherry Y. Leung, and Sandra J. Weiss in Biological Research For Nursing
