# Supplementary material for: Cytokines and Depressive Symptoms Among Adolescents
Source: Biol Res Nurs. 2025 Feb 4;27(3):400–10. doi: 10.1177/10998004251318385 (PMC12144328; doi:10.1177/10998004251318385)
Supplement: Supplemental Material - Cytokines and Depressive Symptoms Among Adolescents [file sj-pdf-1-brn-10.1177_10998004251318385.pdf]

Supplementary Table 1. Linear Regression[a] Model Examining the Associations between Pro-inflammatory Cytokine IL-1β and Depressive Symptoms

	Outcome				
	Depressive Symptoms				
Exposure	Coef	SE	*p*-value	95% CI	β
IL-1β	0.15	0.23	0.520	-0.30 to 0.60	0.05
Sex					
Male	Ref				
Female	-1.39	0.65	0.034	-2.68 to -0.10	-0.19
Age	0.02	0.18	0.921	-0.34 to 0.37	0.01
Ethnicity					
Not Hispanic	Ref				
Hispanic	-0.26	0.76	0.733	-1.76 to 1.24	-0.03
Household Income					
<$75,000	Ref				
$75,000-$100,000	0.001	1.04	0.999	-2.06 to 2.06	0.001
$101,000-$150,000	1.43	0.77	0.065	-0.09 to 2.95	0.19
>$150,000	1.42	1.09	0.193	-0.73 to 3.57	0.11
BMI	-0.04	0.08	0.577	-0.19 to 0.11	-0.05

Note. Abbreviations: Coef, unstandardized beta coefficient; SE, standard error; CI, confidence interval; β, standardized beta coefficient

[a]Adjusted for sex, age, ethnicity, household income, and BMI

Supplementary Table 2. Linear Regression[a] Model Examining the Associations between Pro-inflammatory Cytokine TNF-α and Depressive Symptoms

	Outcome				
	Depressive Symptoms				
Exposure	Coef	SE	*p*-value	95% CI	β
TNF-α	0.19	0.33	0.553	-0.45 to 0.84	0.05
Sex					
Male	Ref				
Female	-1.43	0.65	0.029	-2.72 to -0.15	-0.19
Age	0.02	0.18	0.929	-0.34 to 0.37	0.01
Ethnicity					
Not Hispanic	Ref				
Hispanic	-0.23	0.75	0.759	-1.72 to 1.26	-0.03
Household Income					
<$75,000	Ref				
$75,000-$100,000	0.03	1.05	0.977	-2.04 to 2.10	0.00
$101,000-$150,000	1.43	0.77	0.065	-0.09 to 2.95	0.19
>$150,000	1.40	1.09	0.199	-0.74 to 3.55	0.11
BMI	-0.05	0.07	0.509	-0.20 to 0.10	-0.06

Note. Abbreviations: Coef, unstandardized beta coefficient; SE, standard error; CI, confidence interval; β, standardized beta coefficient

[a]Adjusted for sex, age, ethnicity, household income, and BMI

Supplementary Table 3. Logistic Regression[a] Model Examining the Associations between Pro-inflammatory Cytokine IL-6 and PHQ-9 Depression

	Outcome Depression			
Exposure	OR	SE	*p*-value	95% CI
IL-6	1.13	0.31	0.661	0.66 to 1.93
Age	1.84	0.29	0.001	1.35 to 2.5
Ethnicity				
Not Hispanic	1			
Hispanic	8.30	4.58	0.001	2.82 to 24.45
Household Income				
<$75,000	1			
$75,000-$100,000	0.89	0.77	0.896	0.16 to 4.84
$101,000-$150,000	1.00	0.62	0.999	0.30 to 3.34
>$150,000	0.57	0.55	0.561	0.08 to 3.84
BMI	1.02	0.06	0.798	0.90 to 1.14

Note. Abbreviations: PHQ-9, Patient Health Questionnaire-9; OR, odds ratio; CI, confidence interval

[a]Adjusted for age, ethnicity, household income, and BMI
